# Emerging evidence of coding mutations in the ubiquitin–proteasome system associated with cerebellar ataxias

**DOI:** 10.1038/hgv.2014.18

**Published:** 2014-10-23

**Authors:** Sarah M Ronnebaum, Cam Patterson, Jonathan C Schisler

**Affiliations:** 1 McAllister Heart Institute, The University of North Carolina at Chapel Hill, Chapel Hill, NC, USA; 2 Presbyterian Hospital/Weill-Cornell Medical Center, New York, NY, USA; 3 Department of Pharmacology, The University of North Carolina at Chapel Hill, Chapel Hill, NC, USA

## Abstract

Cerebellar ataxia (CA) is a disorder associated with impairments in balance, coordination, and gait caused by degeneration of the cerebellum. The mutations associated with CA affect functionally diverse genes; furthermore, the underlying genetic basis of a given CA is unknown in many patients. Exome sequencing has emerged as a cost-effective technology to discover novel genetic mutations, including autosomal recessive CA (ARCA). Five recent studies that describe how exome sequencing performed on a diverse pool of ARCA patients revealed 14 unique mutations in *STUB1*, a gene that encodes carboxy terminus of Hsp70-interacting protein (CHIP). CHIP mediates protein quality control through chaperone and ubiquitin ligase activities and is implicated in alleviating proteotoxicity in several neurodegenerative diseases. However, these recent studies linking *STUB1* mutations to various forms of ataxia are the first indications that CHIP is directly involved in the progression of a human disease. Similar exome-sequencing studies have revealed novel mutations in ubiquitin-related proteins associated with CA and other neurological disorders. This review provides an overview of CA, describes the benefits and limitations of exome sequencing, outlines newly discovered *STUB1* mutations, and theorizes on how CHIP and other ubiquitin-related proteins function to prevent neurological deterioration.

## Introduction

‘Ataxia’ is a general term used to describe a loss of coordination. Ataxia may be caused by a variety of diseases, including metabolic disorders, vitamin deficiencies, peripheral neuropathy, cancer, or brain injuries. However, ataxia may also be the result of progressive deterioration of the cerebellum, which can be caused by a huge variety of relatively rare genetic mutations in a family of disorders termed ‘cerebellar ataxia’ (CA). In addition to alterations in movement and balance, CA diseases can be accompanied by impairments in speech, vision, and cognitive ability. The diseases caused by CA mutations are inherited most commonly in autosomal recessive CA (ARCA, estimated prevalence is 7 per 100,000) or autosomal dominant CA (ADCA, estimated prevalence is 3 per 100,000) manners, in addition to less prevalent mitochondrial or X-linked inheritance. Many forms of ADCAs are caused by polyglutamine expansions within a protein-coding region, whereas some ADCAs and most ARCAs are caused by conventional mutations within the coding region (see Table 1). CAs can also manifest as a secondary feature of neurological diseases affecting the brain, such as Huntington disease, Parkinson disease, cerebral palsy, and dentatorubral pallidoluysian atrophy. The age of onset, prognosis, accompanying symptoms, and possible treatment for a given CA depends on the underlying genetic mutation.

Until a few years ago, the rare occurrence and broad clinical heterogeneity of CAs hindered the identification of underlying genetic contributors. Prior to 2010, it had been estimated that the genetic cause was unknown in ~40% of ADCA and ARCA cases.^1^ Identifying the genetic basis of CA is crucial for patients because pinpointing the mutation can lead to information on treatments to help manage the disease, identification of relatives currently at risk of developing the disease, and accurate prenatal counseling for family members. Although helping patients is the foremost priority, genetic identification may also implicate novel biological roles for ataxia-associated genes.

### The advantages and limitations of using exome sequencing to identify rare disease mutations

As whole-genome sequencing gradually becomes more accessible due to lower costs and better data analysis methods, researchers will continue to learn more about how noncoding regions of the genome may regulate processes in a variety of diseases,^2^ including neurological diseases.^3^ However, although the exome (the exon-associated portion of DNA that is transcribed into mature mRNA) represents <1% of the entire genome, the current inventory of disease-causing mutations appear to be disproportionately found in protein-coding regions.^4^ Exome sequencing has emerged as a powerful technology to identify the genetic cause of rare diseases like CAs, and is emblematic of how specialized technology can be used universally within just a few years. First used successfully to identify the basis of a rare disease in 2009,^5^ progressively lower costs and faster sequencing platforms have promoted the mainstream use of exome sequencing over whole-genome sequencing today.

Although exome sequencing currently has many advantages over whole-genome sequencing, exome sequencing has technical limitations beyond the inherent inability to detect mutations in noncoding regions, including difficulty in demonstrating sufficient genetic coverage, a potential inadequacy in identifying chromosomal rearrangements, and difficulty in sequencing trinucleotide repeats, such as the polyglutamine repeats, that characterize the majority of known mutations in ADCA. Still, exome sequencing is well suited for studying rare diseases caused by conventional mutations, including ARCAs. There are several approaches a clinical researcher can take when trying to identify the basis of an ARCA. For example, an analysis of the exome profiles of unrelated individuals presenting with the same syndrome may allow researchers to pinpoint a disease-causing mutation. However, a particular CA may be caused by mutations in different genes or by different mutations within the same gene. A second approach to identifying a disease-causing mutation is to sequence exomes from affected and nonaffected individuals within a multigenerational pedigree, although it is not always possible to obtain a sufficient number of genetic samples. In either approach, a major concern in studying autosomal recessive diseases through sequencing is properly filtering single nucleotide polymorphisms against control databases, such as dbSNP and 1000 Genomes Project, since a ‘control’ population has a high likelihood of expressing a recessive mutation.^6^ In 2011, it was estimated that exome sequencing could identify around 20,000 single nucleotide polymorphisms per genetic sample, of which between 1 and 2% were novel.^7^ As exome-sequencing data for control populations becomes increasingly accessible,^8^ it will be easier to differentiate single nucleotide polymorphismss from novel disease-causing mutations.

### Identification of a STUB1 mutation associated with Gordon Holmes syndrome

**Figure 1:**
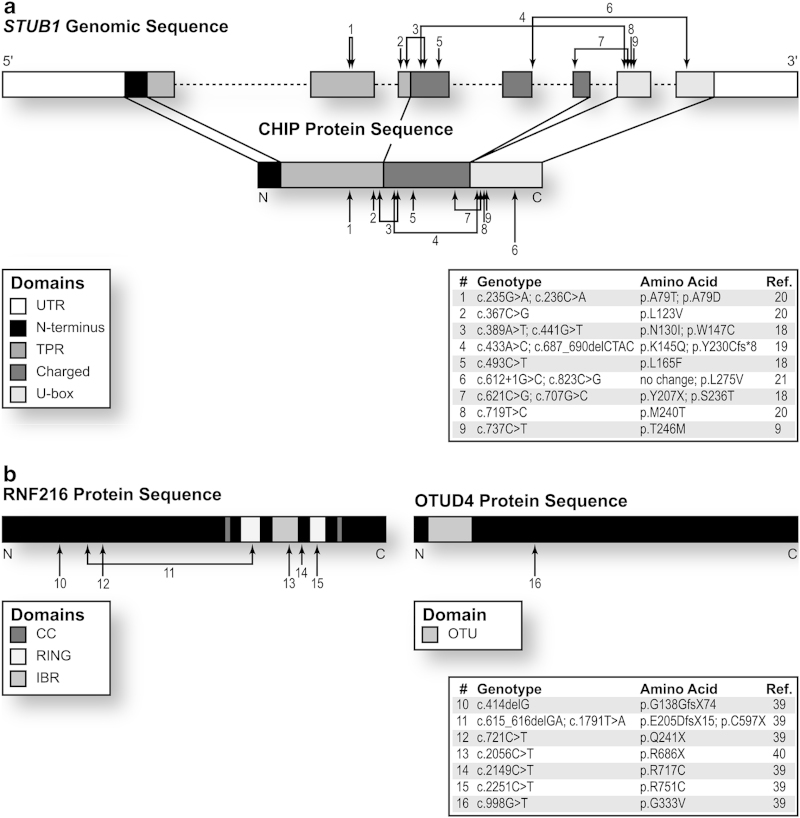
(**a**) *STUB1* genomic structure and corresponding CHIP protein domains are diagramed. The locations (arrows) of the various mutations associated with ARCA and respective nucleic acid and amino acid changes are indicated in the inset table.^9,8–21^ Joined arrows indicate a compound heterozygous mutation. (**b**) The protein structure of RNF216 (left) and OTUD4 (right) are shown with mutations indicated with arrows and identified in the inset table.^39,40^ The domain abbreviations are: UTR, untranslated region; TPR, tetratricopeptide repeat; CC, coiled coil; RING, really interesting new gene; IBR, in-between RING; OTU, ovarian tumor like.

Recently, exome sequencing was used to identify the probable disease-causing mutation in two siblings diagnosed with Gordon Holmes Syndrome (GHS; OMIM #212840), an ARCA characterized by ataxia and hypogonadism.^9^ The exomes of the two affected sisters and an unaffected male sibling were sequenced, and after filtering for novel variants, a recessive inheritance pattern, location within Identical By Decent regions, and predicted effects on protein function, a single homozygous mutation within the coding region of *STUB1* met all criteria in the GHS patients.^9^ Carboxy terminus of Hsp70-interacting protein (CHIP), the protein product of *STUB1*, is a cochaperone and ubiquitin ligase that contains three major protein domains: a tetratricopeptide repeat (TPR) domain required for interaction with heat shock proteins (Hsp), a charged domain that mediates CHIP’s dimerization and activity, and a U-box domain that confers ubiquitin ligase activity (Figure 1a). CHIP forms a homodimer and directly binds to Hsp70 and Hsp90 to aid in refolding substrates, or associates with ubiquitin-conjugating enzymes to ubiquitinate substrates with canonical or noncanonical chains.^10–14^ CHIP can also directly act as an autonomous chaperone by promoting the proper folding and activity of substrates.^15,16^ The homozygous GHS-associated *STUB1* mutation found in this study (c.737C>T) results in the substitution of methionine for threonine at residue 246 (p.T246M) and is located within CHIP’s U-box domain (Figure 1a, Table 2).^9^ *In vitro* studies indicated that the T246M mutation abolishes the ubiquitin ligase activity of CHIP but does not disrupt the association between CHIP and Hsp70.^9^

The GHS patients described in the above study exhibited an unsteady gait that progressed to ataxia, cognitive impairments, and inadequate sexual organ development accompanied by low circulating levels of hormones required for reproductive development (Table 2).^9^ Interestingly, CHIP^−/−^ mice share these phenotypes, although these deficiencies were initially overlooked due to the more prominent findings of acute stress intolerance and premature aging.^11,17^ However, on the basis of the data linking CHIP to GHS, the neurological and reproductive phenotypes of CHIP^−/−^ mice were more closely examined, and impairments in motor activity and spatial learning, cerebellar atrophy, and hypogonadism associated with low circulating hormone levels were found.^9^ The combination of exome sequencing, *in vitro* data, and recapitulation of the GHS phenotype in a mouse model provide high confidence that CHIP, which was previously unknown to have a direct role in any human disease, is essential for cerebellar maintenance.

### Identification of multiple STUB1 mutations associated with ARCA

Bolstering the evidence that loss-of-function mutations in CHIP are causal to ARCA, a second group using exome sequencing reported *STUB1* mutations in six patients with ataxia and cerebellar degeneration from three unrelated families.^18^ In one family, all affected siblings demonstrated a homozygous mutation in *STUB1*, whereas the patients in the remaining families demonstrated compound heterozygous mutations (Figure 1a; Table 2), all of which were predicted to substantially affect CHIP protein function. The *in silico* predictions of altered protein function were corroborated with data demonstrating that these various *STUB1* mutations were associated with a reduction in the degradation of a known CHIP substrate.^18^

A third group recently reported novel *STUB1* mutations in two siblings with ARCA who demonstrate cognitive deterioration.^19^ After ruling out polyglutamine expansions and mutations in over a hundred ataxia-related genes, exome sequencing was employed to determine whether a novel mutation was involved. Compound heterozygous mutations in *STUB1* were discovered in the two patients; these mutations produced an amino-acid substitution in the charged domain and a deletion in the U-box domain that leads to a frameshift mutation, causing the insertion of eight new amino acids and a premature stop codon (Figure 1a; Table 2).^19^ The two patients identified in this study developed symptoms in early adulthood that consisted of gait and speech difficulties, cognitive impairment, and cerebellar atrophy, although the circulating levels of testosterone, luteinizing hormone, and follicle stimulating hormone were normal, in contrast to the patients with the T246M mutation.^19^

In addition, a fourth group performed exome sequencing on a large population of ARCA patients that had been prescreened for mutations in the most common ARCA diseases, and the researchers discovered four novel *STUB1* mutations in three patients.^20^ Two unrelated patients harbored homozygous mutations that led to amino-acid substitutions in the TPR domain and in the U-box domain, whereas the other two patients were siblings and expressed compound heterozygous mutations causing amino-acid substitutions in the same amino acid within the TPR domain (Figure 1a; Table 2).^20^ Interestingly, all patients in this study had normal levels of estrogen and testosterone.^20^ The only patient in this study demonstrating cognitive impairment expressed the M240T mutation found within the U-box.^20^

Most recently, another group has reported on the case of ataxia with myoclonus (muscle jerking), speech difficulties, balance deterioration, and cognitive impairment in a patient harboring compound heterozygous mutations in *STUB1*.^21^ One mutation affected the first base of the intron between the fourth and fifth exons, and the other affected the U-box domain.^21^ Together, these studies demonstrate that mutations in CHIP are causative in multiple ARCA patients from Asian^9,18^ and Caucasian^19–21^ ethnicities, and the cerebellar degeneration may or may not be associated with cognitive impairment and hypogonadism.^9,18–21^ However, judging from the incidence of cognitive impairment occurring in four out of five genetic signatures harboring mutations that affect the U-box (Table 2), it is possible that residual CHIP activity involving an intact TPR domain mitigates clinical symptoms in some patients. At this point, the only available animal model to study CHIP function is the mouse in which the entire *STUB1* gene has been deleted.^11,17^ Although it is difficult to say at this time how mutations within the TRP, charged, or U-box domains specifically affect the symptoms associated with ARCA, the development of animal models with isolated domain mutations may help identify how the bifunctional roles of CHIP affect clinical pathologies.

### The pathophysiological role of CHIP in neurodegenerative diseases

**Figure 2:**
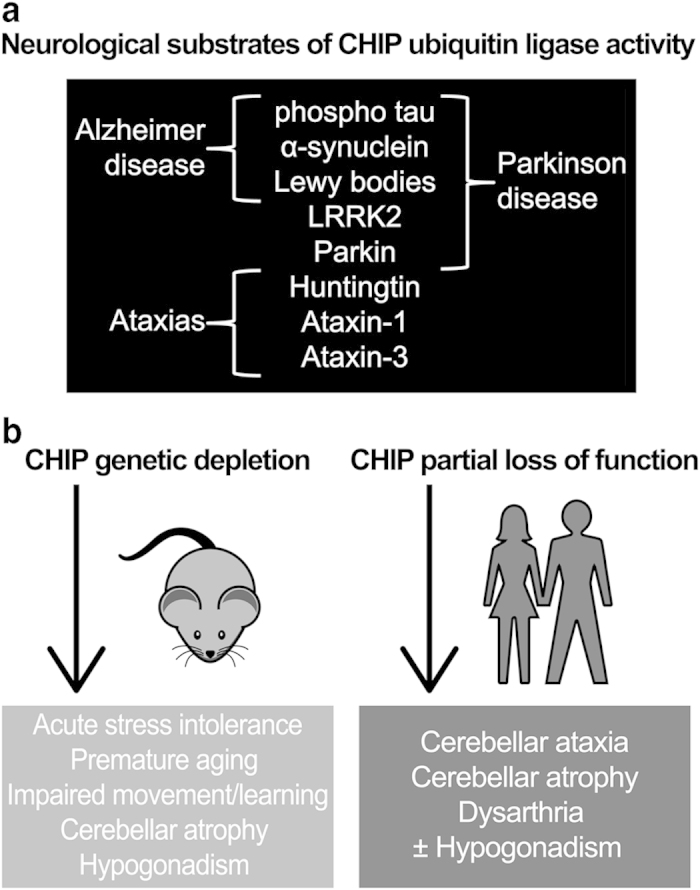
(**a**) The summary of the neurological proteins that serve as CHIP substrates. (**b**) The effects of CHIP genetic depletion from mouse models (left) and observed effects on humans with CHIP loss-of-function mutations (right).

Models of neurodegenerative diseases previously identified a plausible role for CHIP in regulating neurological function; however, not until the recent clinical studies has there been any evidence that loss-of-function mutations in CHIP lead to severe CA disease phenotypes in humans.^9,18–21^ CHIP associates with numerous neuronal proteins. For example, CHIP is detected in Lewy bodies;^22^ and CHIP recognizes and clears phosphorylated tau^23^ and α-synuclein.^22,24^ CHIP suppresses toxicity caused by LRRK2^25,26^ and Huntingtin,^27,28^ and enhances the ubiquitin ligase activity of wild-type Parkin.^29^ In a direct link to an ADCA, CHIP mediates the degradation of a mutated polyglutamine-expanded ataxin-1, a causative mutation in spinocerebellar ataxia 1 (SCA1).^30^ CHIP also participates in a negative feedback cycle with the E2 ubiquitin-conjugating enzyme E2W (Ube2w)and ataxin-3,^31^ a DUB that targets noncanonical chains.^32,33^ Similar to ataxin-1, a polyglutamine-expansion mutation in ataxin-3 is implicated in the ADCA SCA3,^34^ and the severity of the pathological phenotype of transgenic mice expressing the ataxin-3 polyglutamine-repeat mutation is inversely proportional to CHIP copy number expression status.^35^ It should be noted that the role of CHIP in mitigating mutant ataxin-3 toxicity in other ataxin-3 mutation models is unclear.^36^ However, it is unlikely that the proteins listed above are implicated in the pathology of the ARCA patients expressing *STUB1* mutations, as there were no additional mutations found.^9,18–21^ Supportive of the notion that loss of CHIP alone directly causes ARCA is the observation that the early-onset of disease and distinctive pathology of ARCA described in these recent studies^9,18–21^ does not coincide with symptoms of SCA1, SCA3, Parkinson, Alzheimer, or Huntington diseases. Together, these data support a model where CHIP is involved in mediating disease progression when a client protein is mutated; however, it is now clear that a loss of CHIP function, caused by either substitution or truncation mutations (Figure 1a), also leads to a severe pathological consequence through an as yet undetermined mechanism (summarized in Figure 2).

### Mutations in the ubiquitin ligase RNF216 and the deubiquitianse OTUD4 also associate with GHS

Of the 13 exon mutations in CHIP found in ARCA patients, 9 affect either the charged linker region or the U-box domain (Figure 1a), which are domains required for CHIP function and/or dimerization.^37,38^ The identification of ataxia-associated mutations that abolish CHIP’s ubiquitination activity,^9,18–21^ in addition to recent reports of exome mutations in other proteins involved in ubiquitination in other ataxia-related conditions,^39,40^ support the distinct connection between neurological diseases and defects in the ubiquitin–proteasome system (UPS) (reviewed in references 41–44). A recent study of three siblings with GHS used exome sequencing to find homozygous mutations in both the ubiquitin ligase ring finger protein 216 (*RNF216*) and the deubiquitinase OTU domain containing 4 (*OTUD4*), and sequencing of *RNF216* in six unrelated GHS patients demonstrated a variety of heterozygous *RNF216* mutations (Figure 1b).^39^ Importantly, the authors verified the involvement of *RNF216* and *OTUD4* in cerebellar function using zebrafish models demonstrating how the depletion of either gene significantly increases the appearance of cerebellar defects.^39^ Subsequently, an exome-sequencing study consisting of several ataxia patients identified a unique *RNF216* mutation in a GHS patient (Figure 1b).^40^ Unlike CHIP, targets of RNF216 ubiquitin ligase activity are not well characterized. However, overlap in the GHS phenotype associating with CHIP and RNF216 mutations affords a unique comparative opportunity to study CHIP and RNF216 activities that may provide the molecular starting point to determine the mechanism by which the loss of either E3 ligase could lead to GHS.

### CA pathologies—converging at protein quality control?

The diverse etiology of the CA phenotype presents a challenge to researchers. One common feature of some proteins involved in ADCAs is polyglutamine expansion; otherwise, most ADCA proteins are functionally unrelated (Table 1). As discussed above, mutations in different genes such as *STUB1* and *RNF216* can give rise to a similar phenotype. When the causal genes are functionally related, it may provide some insight into the pathophysiology. However, there are instances where causal genes of a shared phenotype do not appear to be functionally related; for example, Boucher–Neuhäuser Syndrome (OMIM #215470) and GHS are both early-onset ARCAs accompanied by hypogonadotropic hypogonadism. Although Boucher–Neuhäuser Syndrome is characterized by chorioretinal dystrophy and GHS by brisk reflex, both diseases can be caused by mutations in patatin-like phospholipase domain containing 6 (*PNPLA6*),^45^ an enzyme with little functional overlap with CHIP or RNF216. It is easy to hypothesize that PNPLA6 is a substrate for either CHIP or RNF216, but there has yet to be any data published to support this, and currently it is unclear how PNPLA6 may intersect with the UPS. However, in addition to the longstanding observation that ubiquitin is present in aggregates that are hallmarks of several neurodegenerative diseases,^46^ the use of exome sequencing in recent studies demonstrated that multiple components of the UPS are involved in CAs and other neurological and neurodegenerative disorders. For example, ubiquitin carboxyl-terminal esterase L1 (*UCHL1*), a deubiquitinating enzyme that may also have ubiquitin ligase activity, has previously been linked to Parkinson and Alzheimer diseases.^47,48^ A homozygous missense mutation in *UCHL1* was found in three siblings with ataxia and severe cerebellar atrophy,^49^ similar to mouse models in which *UCHL1* is mutated or deleted.^50–52^ Similarly, a mutation in ubiquitin protein ligase E3 component N-recognin 4 (*UBR4*) was detected in a family with episodic ataxia through exome sequencing.^53^ UPS components have also been found to be mutated in some familial cases of amyotrophic lateral sclerosis, including ubiquilin 2, a ubiquitin-like protein,^54,55^ and valosin-containing protein (*VCP*), a ubiquitin segregase.^56^ Exome sequencing also identified two different mutations in an under-characterized protein, ubiquitin protein ligase E3B (*UBE3B*), associated with Kaufman Oculocerebrofacial Syndrome,^57^ whereas other *UBE3B* mutations result in an intellectual deficiency disorder^58^ or associate with autism,^59^ again demonstrating how distinct mutations in the same gene can give rise to different phenotypes. In addition, some ARCAs are attributed to mutations in genes involved in the UPS or in the chaperoning of proteins (Table 1). One only has to look at the myriad known CHIP substrates (Figure 2a) and phenotypes from CHIP loss-of-function studies (Figure 2b) to appreciate the neuronal demand for protein quality control. It is not surprising that a collective theme is emerging, at least within a subset of CAs, that clearly has a direct genetic link to the UPS and protein quality control. Perhaps other known CA casual mutations in genes not directly associated with the UPS cause a change in protein stability, or perhaps participate in signaling networks that intersect the UPS and protein quality control pathways in mechanisms not previously characterized. Future efforts focusing on the molecular characterization of newly identified coding mutations associated with CAs should consider effects on protein stability and possible implications to the UPS, as protein quality control is paramount in maintaining neuronal cell homeostasis.

## Conclusions

As the global access to exome-sequencing technology and analysis becomes increasingly available, there is tremendous potential for successful identification of causal mutations of rare diseases, such as CAs. The clinical identification of these mutations, combined with basic and translational research approaches, will foster new insights into human diseases and uncover novel roles for genes and proteins that have direct links to human diseases. We are confident that collaborative studies combining clinical identification of mutations and basic research models validating gene and protein function will uncover the molecular mechanisms by which a single mutation leads to a devastating condition and will foster therapies to help patients with rare genetic diseases.

## Figures and Tables

**Table 1:** A summary of se veral CA subtypes, associated OMIM number, mutated genes, and function of the affected protein

*Disease*	*OMIM*	*Gene*	*Protein function*
ADCAs			
*Repeat expansions in exons*			
DRPLA	125370	ATN1	Transcriptional corepressor
HD	143100	HTT	Microtubule-mediated vesicle transport
HDL1	603218	PRNP	Copper iron; microtubule binding
HDL2	606438	JPH3	Calcium-release channel activity
SCA1	164400	ATXN1	Nucleic acid binding
SCA17/HDL4	607136	TBP	Transcription factor
SCA2	183090	ATXN2	RNA binding
SCA3	109150	ATXN3	Deubiquitinating enzyme
SCA6	183086	CACNA1A	Voltage-gated calcium channel
SCA7	164500	ATXN7	Chromatin binding
*Repeat expansions in noncoding regions*			
SCA10	603516	ATXN10	Unknown
SCA12	604326	PPP2R2B	Protein phosphatase
SCA31	117210	BEAN1	Unknown
SCA36	614153	NOP56	Ribosomal RNA subunit biogenesis
SCA8	608768	ATXN8OS	Noncoding gene
*Missense, nonsense, insertion, or deletion mutations*			
ADCA-DN	604121	DNMT1	DNA methyltransferase
CANPMR	614756	CAMTA1	Transcriptional activator
Cortical myoclonus	614937	NOL3	RNA binding
Familiar dementia	176500	ITM2B	ATP; beta amyloid binding
Gillespie syndrome	206700	PAX6	Transcription factor
SCA5	600224	SPTBN2	Cytoskeleton component
SCA11	604432	TTBK2	Protein kinase
SCA13	605259	KCNC3	Voltage-gated potassium channel
SCA14	605361	PRKCG	Calcium-dependent protein kinase
SCA15/SCA16	606658	ITPR1	Ligand-gated calcium channel
SCA19/SCA22	607346	KCND3	Voltage-gated potassium channel
SCA23	610245	PDYN	Neuropeptide hormone activity
SCA26	609306	EEF2	Translation factor
SCA27	609307	FGF14	Growth factor
SCA28	610246	AFG3L2	ATP-dependent protease
SCA35	613908	TGM6	Protein crosslinking; polyamine conjugation
			
ARCAs			
AOA1	208920	APTX	DNA repair
AOA2	606002	SETX	DNA/RNA helicase
AOA3	615217	PIK3R5	PI3K regulation
Ataxia telangectasia	208900	ATM	DNA damage-dependent protein kinase
Ataxia telangectasia-like disorder	604391	MRE11A	DNA repair
Cayman	601238	ATCAY	Unknown
Charlevoix-Saguenay	270550	SACS	Cochaperone
Cockayne syndrome A	216400	ERCC8	DNA repair; ubiquitin ligase complex component
Cockayne syndrome B	133540	ERCC6	DNA repair
Coenzyme Q10 deficiency	607426	COQ2	Coenzyme Q biosynthesis
Friedreich ataxia	229300	FXN	Iron homeostasis
Marinesco–Sjogren	248800	SIL1	Protein translocation/folding
MGA5	610198	DNAJC19	Protein translocation; cochaperone
PHARC	612674	ABHD12	Lipid hydrolysis
SCAN1	607250	TDP1	DNA repair
SCAR8	610743	SYNE1	Cytoskeleton
SCAR9	612016	CABC1	Kinase; coenzyme Q biosynthesis

Abbreviations: ADCAs, autosomal dominant cerebellar ataxias; ARCAs, autosomal recessive cerebellar ataxias; CA, cerebellar ataxia; DRPLA, dentatorubral pallidoluysian atrophy; HD, Huntington disease; SCA, spinocerebellar ataxia.

**Table 2:** The affected protein domains, mutation type, and clinical phenotypes of the patients with *STUB1* mutations as indicated in Figure 1a are provided^9,18–21^

*#*	*Affected domain (s)*	*Type*	*Tendon reflex*	*Cognitive impairment*	*Hypogonadism*	*Reference*
1	TPR	CHet	Increased	No	No	^20^
2	TPR	Hom	Normal	No	No	^20^
3	TPR; charged	CHet	Normal	No	Not reported	^18^
4	Charged; U-box	CHet	Increased	Severe	No	^19^
5	Charged	Hom	Normal/increased	Normal/mild	Not reported	^18^
6	Intron; U-box	CHet	Increased	Severe	Not reported	^21^
7	Charged; U-box	CHet	Increased	Normal	Not reported	^18^
8	U-box	Hom	Normal	Severe	No	^20^
9	U-box	Hom	Normal/increased	Severe	Yes	^9^

‘CHet’ and ‘Hom’ refer to compound heterozygous and homozygous mutations, respectively.

Abbreviation: TPR, tetratricopeptide repeat.
